# Items

**Published:** 1880-10

**Authors:** 


### Items.

LIST OF BOOKS WHICH WILL BE FOUND VALUABLE TO CANDIDATES FOR EXAMINATION IN SANITARY SCIENCE BY THE MICHIGAN STATE BOARD OF HEALTH.

The books are here classified the same as the topics published in the Regulations for the Examinations. The necessity of reading all of the books is not urged.

I.—Introduction to the study of Biology. H. Alleyns Nicholson. Appleton & Co., New York, 75 cents. Article on Biology in Encyclopædia Britannica and in other Cyclopædias.

II.—(*a.*) Theory of Probabilities. M. A. Quetelet, translated by O. G. Downs. C. & E. Layton, London, England, $5.50. Articles on Statistics and Vital Statistics in various cyclopædias. Essays and Papers on Some Fallacies of Statistics. H. W. Rumsey. Smith, Elder & Co., London, England, 12 shillings. Vital Statistics of Michigan, Annual Reports 1868–1874. Article—Report on Methods of Collection of Vital Statistics in Report of Michigan State Board of Health for 1876. Article on "Weekly Reports of Diseases in Michigan." Annual Reports Michigan State Board of Health, 1878–1880. (*b.*) Vital Statistics of Michigan for 1872 and subsequent years. Annual Reports of the Michigan State Board of Health, 1878–1880. Articles on "Weekly Reports of Diseases," and "Principal Meteorological Conditions in Michigan." (*c.*) Filth Diseases and their Prevention. John Sineon, M.D. James Campbell, Boston, Mass. Disposal of Slop Water in Villages. C. B. Fox J. & A. Churchill, London, England, 18d. Healthy Houses. Fleming Jenkin, F.R.S., adapted to American Conditions by George E. Waring, Jr. Harper's Half-Hour Series, 25 cents. House Drainage and

Water Service. James C. Bayles. David Williams, New York, $3.00. Wilson's Handbook of Hygiene. Blakiston, Philadelphia, $3.00. Hart's Manual of Public Health. Smith, Elder & Co., London, England, 12s. 6d. (*d.*) Anstie on Stimulants and Narcotics. Blakiston, Philadelphia, Penn., $3.00. Tables, etc., on Mortality by Occupations, in United States Census, and in Vital Statistics of Michigan, 1868–1874. (*e.*) Disease Germs; Their Nature and Origin. L. S. Beale. Blakiston, Philadelphia, $5.00. The Germ Theory of Disease. Mælagan. Macmillan & Co., London, England, $3.00. *Lectures on the Theory and General Prevention and Control of Infectious Diseases, by Jas. B. Russell. James Maclehose, Glasgow, Scotland. Fermentation. Schützenberger. Appleton & Co., New York, $1.50. Practical Biology. Huxley and Martin. Macmillan & Co., London, $1.50. Air and its Relations to Life. W. N. Hartley. D. Appleton & Co., New York, $1.50. Article on Origin and Propagation of Disease. John C. Dalton, M.D., read before the New York Academy of Medicine. Smithsonian Report, 1873. (*f.*) *Lectures on the Theory and General Prevention and Control of Infectious Disease. Jas. B. Russell. James Maclehose, Glasgow, Scotland. (*g.*) Parkes' Hygiene, edited by De Chaumont, Blakiston, Philadelphia, $6.00. Documents on "Restriction and Prevention of Scarlet Fever;" on "Restriction and Prevention of Diphtheria;" Circulars 34 and 35; issued by the Michigan State Board of Health, Lansing. *Lectures on the Theory and General Prevention and Control of Infectious Diseases, by Jas. B. Russell. James Maclehose, Glasgow, Scotland. Annual Reports of the Michigan State Board of Health, 1876–1880.

III.—(*a.*) Attfield's General Medical and Pharmaceutical Chemistry, 8th Revised ed. Henry C. Lea's Son & Co., Philadelphia, Penn., $2.50. *Lectures on Air, Water Supply, Sewage Disposal, and Food. William Wallace. James Maclehose, Glasgow, Scotland. Letheby on Food. Wm. Wood & Co., New York, $2.25. Hassells' Food and its Adulterations. Longmans, Green & Co., England. Parkes' Hygiene, edited by De Chaumont, Blakiston, Philadelphia, $6.00. Wanklyn and Chapman's

---

*The lectures by James B. Russell, M.D., and William Wallace, are published in one volume by James Maclehose, Glasgow, Scotland; price, 1s. Adams and Co., London, England.

Water Analysis. Trübner, London, England, $2.50. Frankland's Water Analysis for Sanitary Purposes. Blakiston, Philadelphia, $1.00. (*b.*) Loomis on Meteorology. Harper, New York, $1.75. (For reference.) Air and Rain. R. Angus Smith. Longmans, Green & Co., London, 34s. Annual Reports of Michigan State Board of Health for 1874, article on "Meteorology of Central Michigan;" 1875, directions for taking meteorological observations, also an article on Ozone; 1878–1880, articles on "Principal Meteorological Conditions in Michigan." Air and its Relations to Life. W. N. Hartley. D. Appleton & Co., New York, $1.50.

IV.—Sanitary Engineering. Baldwin Latham. E. and F. Spon, London, England. Sanitary Engineering. J. Bailey Denton. E. & F. Spon, London, England. Parkes' Practical Hygiene, edited by De Chaumont. Blakiston, Philadelphia, $6.00. House Drainage and Water Service. James C. Bayles. David Williams, New York, $3.00. The House and its Surroundings. D. Appleton & Co., New York, 40 cents. Sanitary Houses. Two Lectures, by James A. Russell, A.M., M.B., etc. Maclachlan and Stewart, Edinburgh. Simpkin, Marshall and Co., London, England. Sanitary Work in Towns and Villages. Charles Slagg. Crosby, Lockwood & Co., London, England. Sanitary Work in Villages and Country Districts. George Wilson. J. & F. Churchill, London, England, 18d. *Lectures on Air, Water Supply, Sewage Disposal, and Food. William Wallace. James Maclehose, Glasgow, Scotland. Healthy Houses. Wm. Eassie. D. Appleton & Co., New York, $1.00.

V.—House Drainage and Water Service. James C. Bayles. David Williams, New York, $3.00. Handbook for Inspectors of Nuisances. Edward Smith, M.D. Knight & Co., London, England, 5s. *Lectures on the Theory and General Prevention and Control of Infectious Diseases. James B. Russell. James Maclehose, Glasgow, Scotland. The Sewage Question—with special reference to Traps and Pipes. H. Fergus, M.D., M.R.C.S. Porteus Brothers, Glasgow, Scotland, 18d. Annual Reports of the Michigan State Board of Health, 1873–1880. Schedule for Sanitary Survey of a City. Am. Public Health Association

---

* The lectures by James B. Russell, M.D., and William Wallace, are published in one volume by James Maclehose, Glasgow, Scotland; price, 1s. Adams and Co., London, England.

Published by U. S. National Board of Health, Washington, D.C. Sanitary Inspection of Memphis, Tennessee, Schedules and Report. National Board of Health, Washington, D. C.

VI.—Compiled Laws of Michigan, 1871. Public Health Enactments in Session Laws of Michigan since 1871, particularly late ones, 1877, 1879 and 1881 (?), with index of laws amended, etc. Public Health Laws of Michigan. Pamphlet, 1876, State Board of Health, Lansing. Postage, 3 cents.

In all cases where known, the price of the book, and the name of the American publisher has been given.

HENRY B. BAKER, *Secretary.*

OFFICE OF STATE BOARD OF HEALTH, LANSING, MICH., July 30, 1880.

OFFICIAL LIST OF CHANGES OF STATIONS AND DUTIES OF MEDICAL OFFICERS OF THE MARINE HOSPITAL SERVICE OF THE UNITED STATES. April 1, 1880, to June 30, 1880.

BAILHACHE, P. H., Surgeon. Detailed as chairman of board for the physical examination of officers of the Revenue Marine Service, April 28, 1880. Detailed as chairman of board for the physical examination of candidates for appointment as cadets in the Revenue Marine Service, May 21, 1880. Detailed as medical officer Revenue Bark "Chase" during practice cruise, June 1, 1880.

MILLER, T. W., Surgeon. Detailed as chairman board of examiners to convene in New York, June 21, 1880, June 4, 1880.

LONG, W. H., Surgeon. Granted leave of absence for ten days from April 16, 1880, April 14, 1880. Detailed as member of board to select a site for a Marine Hospital at Memphis, Tennessee, May 12, 1880.

FESSENDEN, C. S. D., Surgeon. Detailed as member board of examiners to convene in New York, June 21, 1880, June 4, 1880. Granted leave of absence for eight days from June 13, 1880, June 9, 1880.

SAWTELLE, H. W., Surgeon. Detailed as recorder of board to select a site for a Marine Hospital at Memphis, Tennessee, May 12, 1880.

DOERING, E. J., Surgeon. Detailed as recorder board of examiners to convene in New York, June 21, 1880, June 4, 1880.

FISHER, J. C., Passed Assistant Surgeon. Granted leave of absence for thirty days from May 6, 1880, April 21, 1880. Detailed as recorder of board for the physical examination of officers of the Revenue Marine Service, April 28, 1880.

GODFREY, JOHN, Assistant Surgeon. To report to board of examiners for examination for promotion, June 4, 1880.

BROWN, F. H., Assistant Surgeon. To act as inspector of unserviceable hospital property at Boston, Mass., April 13, 1880. To report to board of examiners for examination for promotion, June 4, 1880.

GOLDSBOROUGH, C. B., Assistant Surgeon. Detailed as recorder of board for the physical examinination of candidates for appointment as cadets in the Revenue Marine Service, May 21, 1880.

KEYES, H. M., Assistant Surgeon. To act as inspector of unserviceable hospital property at St. Louis, Mo., April 13, 1880.

MEAD, F. W., Assistant Surgeon. To act as inspector of unserviceable hospital property at San Francisco, Cal., April 19, 1880.

PORTER, F. D., Assistant Surgeon. Granted leave of absence for fourteen days from July 2, 1880, June 29, 1880.

PROMOTION.

FISHER, J. C., Passed Assistant Surgeon. Promoted to be Passed Assistant Surgeon, April 2, 1880.

AN irreparable loss is that sustalned by the Vienna school and the profession of medicine the world over, in the death of Professor Hebra. As an author, clinicien, and teacher of dermatology, he stood without a rival, and his numerous pupils scattered throughout all countries, must now promote that work which the great master only abandoned with his life. He was buried in the Hemals cemetery, in a grave not far from that of his old friend Rokitansky. Prof. Heschl and other eminent gentlemen made eloquent addresses at the funeral ceremonies.

